# Impacts of soybean agriculture on the resistome of the Amazonian soil

**DOI:** 10.3389/fmicb.2022.948188

**Published:** 2022-09-09

**Authors:** Oscar Cardenas Alegria, Marielle Pires Quaresma, Carlos Willian Dias Dantas, Elaine Maria Silva Guedes Lobato, Andressa de Oliveira Aragão, Sandro Patroca da Silva, Amanda Costa Barros da Silva, Ana Cecília Ribeiro Cruz, Rommel Thiago Jucá Ramos, Adriana Ribeiro Carneiro

**Affiliations:** ^1^Laboratory of Genomic and Bioinformatics, Center of Genomics and System Biology, Institute of Biological Sciences, Federal University of Pará, Belém, Brazil; ^2^Institute of Biological Sciences, Federal University of Minas Gerais, Belo Horizonte, Brazil; ^3^Department of Soil Science, Federal Rural University of the Amazon, Paragominas, Brazil; ^4^Department of Arbovirology and Hemorrhagic Fevers, Evandro Chagas Institute-IEC/SVS/MS, Ananindeua, Brazil

**Keywords:** antibiotic resistance genes, biocide resistance genes, heavy metals, metagenomic, Amazonia soils

## Abstract

The soils of the Amazon are complex environments with different organisms cohabiting in continuous adaptation processes; this changes significantly when these environments are modified for the development of agricultural activities that alter the chemical, macro, and microbiological compositions. The metagenomic variations and the levels of the environmental impact of four different soil samples from the Amazon region were evaluated, emphasizing the resistome. Soil samples from the organic phase from the different forest, pasture, and transgenic soybean monocultures of 2–14 years old were collected in triplicate at each site. The samples were divided into two groups, and one group was pre-treated to obtain genetic material to perform sequencing for metagenomic analysis; another group carried out the chemical characterization of the soil, determining the pH, the content of cations, and heavy metals; these were carried out in addition to identifying with different databases the components of the microbiological communities, functional genes, antibiotic and biocide resistance genes. A greater diversity of antibiotic resistance genes was observed in the forest soil. In contrast, in monoculture soils, a large number of biocide resistance genes were evidenced, highlighting the diversity and abundance of crop soils, which showed better resistance to heavy metals than other compounds, with a possible dominance of resistance to iron due to the presence of the *acn* gene. For up to 600 different genes for resistance to antibiotics and 256 genes for biocides were identified, most of which were for heavy metals. The most prevalent was resistance to tetracycline, cephalosporin, penam, fluoroquinolone, chloramphenicol, carbapenem, macrolide, and aminoglycoside, providing evidence for the co-selection of these resistance genes in different soils. Furthermore, the influence of vegetation cover on the forest floor was notable as a protective factor against the impact of human contamination. Regarding chemical characterization, the presence of heavy metals, different stress response mechanisms in monoculture soils, and the abundance of mobile genetic elements in crop and pasture soils stand out. The elimination of the forest increases the diversity of genes for resistance to biocides, favoring the selection of genes for resistance to antibiotics in soils.

## Introduction

The use of land for agriculture has grown proportionally with the world population over time, increasing food production and the areas of cultivation. Among the widely developed cultures, the one with the most significant demand due to its application in different processes of the food industry is the soybean crop, with Brazil being the world's largest producer of soybeans, accounting for more than a third of production—followed by the United States and China (Voora et al., 2020).

In the northern region of the Brazilian territory, there are the most extensive areas of tropical Amazon forest. In the case of the state of Pará, it presents different ecological characteristics of forest, pasture, and the development of cassava, oil palm, soybean, corn, and others (Toloi et al., 2021). The expansion of agricultural frontiers makes it possible to modify the ecosystem by eliminating plant cover and applying chemical compounds, such as fertilizers, pesticides, antibiotics, salts, and others (Basso et al., 2021).

On the other hand, the soil is one of the largest and most diverse microbial habitats, having a vast reservoir of antibiotic resistance genes. Approximately 80% of soil microorganisms resist multiple antimicrobials, so these genes are naturally present in different ecological environments (Berg and Martinez, 2015; Chen et al., 2019). From this perspective, using fertilizers in the soil positively correlates with changing microbial composition and increases the abundance of resistance genes (Udikovic-Kolic et al., 2014; Sun et al., 2019). Thus, these environmental changes increase horizontal genetic transfer in microorganisms and accelerate adaptation to different stress mechanisms (Bottery et al., 2021). The antibiotic resistance genes (ARG) identified in the soil were tetracycline, fluoroquinolones, sulfonamides, β-lactams, aminoglycosides, erythromycin, and others (Jutkina et al., 2018; Armalyte et al., 2019; Brevik et al., 2020).

In this scenario, due to the effects of chemical and organic fertilizers, soil microbial communities are modified and become different from the original communities (Li et al., 2018; Chen et al., 2019). Soil microbial composition is complex and dynamic, as there are cooperative and competitive relationships within the same population, which can strengthen the diversity and stability of the environment (Yang et al., 2018; Qu et al., 2019). Furthermore, they may favor the co-selection of genes associated with adaptation, such as genes associated with resistance to antibiotics, heavy metals, biocides, and detergents, among others (Destoumieux-Garzón et al., 2018; Durso and Cook, 2019; Brevik et al., 2020). Natural levels of biocides such as heavy metals vary according to the type of soil (Costa et al., 2017), as well as cadmium (Cd), zinc (Zn), nickel (Ni), and mercury (Hg) are involved in the co-selection processes of multidrug-resistant bacteria (Heydari et al., 2022). Additionally, in the presence of horizontal transfer genes (HGT), the transfer of these genes is influenced by different abiotic and biotic factors (Hu et al., 2017; Jutkina et al., 2018).

While resistance and tolerance are considered properties of a population, interspecies interactions can have a significant effect on antibiotic and biocide treatment: communities or subpopulations of bacterial communities can survive exposure to antimicrobials due to interactions between species, and various mechanisms can act simultaneously within a community, many of which are widely known, such as antibiotic inactivation, biofilm formation, and quorum sensing activated responses (Bottery et al., 2021). In the case of the toxic effect of heavy metals, many microorganisms have developed efficient mechanisms of resistance to biocides, configuring three main types of proteins normally involved with flow pumps: (i) resistance-nodulation-cell division proteins (RND superfamily), (ii) cation diffusion facilitators (CDF family), and (iii) P-type ATPases (Romaniuk et al., 2018).

Otherwise, various biotic and abiotic stressors can affect agricultural productivity and crop yields, eventually leading to global food shortages (Bhagat et al., 2021). The need to adopt environmentally friendly methods to combat these stressors and improve crop yields is increasingly accentuating due to current agricultural practices' negative impacts (Salam et al., 2020). Plant health is closely linked to soil health, both of which are influenced by microbial communities, which significantly impact agricultural productivity and human health (Altier and Abreo, 2020). Thus, it is currently inevitable to associate human-mediated loss of biodiversity with the transformation of natural ecosystems and how they affect human health (Cook et al., 2020).

The monoculture production model, such as soybean production, requires extensive land use, a high level of mechanization, and intensive use of pesticides—compromising the environment and human health (Belo et al., 2012; Pignati et al., 2017; Basso et al., 2021). Likewise, bacteria usually coexist in complex communities in the soil and present different interactions—including competition, exploitation, commensalism, and mutualism—these respond to exposure to different biotic and abiotic factors, which can alter the strength of selection acting on resistance mechanisms (Bottery et al., 2021), for example, soil pH and heavy metal pollution change the composition of bacterial communities (Chodak et al., 2013). Likewise, gene transfer processes can increase resistance in the different pathogenic organisms that reside in the soil, emphasizing the resistome's clinical importance (Forsberg et al., 2012).

The metagenomic analysis makes it possible to accurately describe the composition of microbial communities and the resistome genes under the environment's selective pressure and the effect of the alteration of ecosystems by agricultural production (Durso and Cook, 2019). In this context, we aimed (i) to evaluate the chemical characteristics of soils with and without tillage, which were exposed to agricultural compounds, (ii) to establish the relationship between soils used for tillage and changes in soil bacterial communities, and (iii) to elucidate the influence of soil properties on the disposition of genes associated with antibiotic and biocide resistance in soils in the Amazon region with different cultures and management times.

## Materials and methods

### Description of the study sites

The study site is in the Amazon, Paragominas-PA, Brazil. The area has been explored for 35 years for intensive grain farming and beef cattle. The predominant soil classified as Oxisol (Rodrigues et al., 2003) and the Aw climate (Alvares et al., 2013), hot and humid tropical, and was divided into four collection sites, being secondary forest (lat 2°58'34.46”S; long 47°18'0.72”W), Patagem (lat 2°58'52.88”S; long 47°18'1.332”W), soybean crop (Glycine max, P98 YH51 RR) 2 years (lat 2°59'2.54”S; long 47°17'18.77”W) and a 14-years-old soybean crop (lat 3°0'3.07”S; long 47°16'59”W). The soils were covered with native tropical forest, but after commercial timber was removed around 30 years ago, these were used for planting transgenic soybean crops and pasture; limestone was used, and chemical fertilizers were added to the cultivated sites [phosphate mono ammonium (MAP), potassium chloride (KCl), and Super triple phosphate]; herbicides; fungicides, and insecticides. As for the 14-years-old monoculture soil, ~20 years ago, it was used for cultivation; at first, rice (*Oryza sativa*) was sown, and later three consecutive crops of corn (*Zea mays*) were used only for soybean cultivation. Samples were collected at a depth of up to 30 cm of soil (topsoil; Bach et al., 2018), and the collection was carried out in triplicate for each type of forest soils, pastures, and places where soybeans were developed for 2–14 continuous years. The collected samples were separated for chemical characterization and molecular analysis. Samples for chemical analysis were stored in 500 g plastic bags and kept at 4°C, whereas for molecular evaluation, the samples were stored in falcon tubes with a capacity of 15 ml and deposited immediately in liquid nitrogen.

### Soil chemical characterization

Raij et al. (2001) described the methodology used for chemical characterization. The samples were dehydrated in an oven with air circulation at 40°C and then sieved with a 2 mm mesh sieve. The pH was determined with water. The micronutrient contents of boro (B), copper (Cu), iron (Fe), manganese (Mn), cadmium (Cd), lead (Pb), cobalt (Co), mercury (Hg), nickel (Ni), and zinc (Zn) were determined by Inductively Coupled Plasma—Atomic Emission Spectrometry (ICP-AES, Thermo Fisher ICAP 7600). The cations were extracted with the chloride solution by the ion exchange process, using the EDTA titration method for the determination of calcium (Ca^2+^), magnesium (Mg^2+^), and potassium (K^+^) cations.

### DNA isolation

The collected soil samples were first treated with sodium phosphate buffer solutions (500 mM Na_2_HPO_4_ and 500 mM NaH_2_PO_4_; pH 7.2), according to the protocol of Högfors-Rönnholm et al. (2018). Then, genetic material was extracted with the Dneasy PowerSoil DNA Isolation Kit (Qiagen, USA). DNA concentrations were measured by a NanoDrop spectrophotometer (Thermo Scientific), and quality was determined by horizontal electrophoresis in a 1% agarose gel with 0.5 ug ul^−1^ of ethidium bromide.

### Next-generation sequencing

For the construction of the DNA libraries, the samples were processed in compliance with the manufacturer's protocol, Nextera XT DNA Library (Illumina). Sequencing was performed with the NextSeq 550 System High-Output kit, generating paired readings of 150 bp using the Illumina NextSeq 500/550 HighOutput platform.

The quality of the reads was analyzed using the FASTQC-Version 0.11.9 software (Andrew, 2019), later trimmed and filtered with a minimum quality standard of Phred 20 and a minimum size of 36 bp by the Trimmomatic software (Bolger et al., 2014).

### Microbial diversity analysis

Data in triplicates were analyzed by Kraken2 (Wood et al., 2019) to identify operational taxonomic units (OTU) and use the Pavian platform (Breitwieser and Salzberg, 2020) to perform diversity analysis. Then, Shannon's alpha diversity indices were calculated using the MicrobiomeAnalyst program (Dhariwal et al., 2017).

### Resistome assembly and analysis

Data assembly was performed using the Megahit program (Li et al., 2015), with a “meta-large” pattern, and the resulting contigs were analyzed using the Metaquast software (Mikheenko et al., 2016). Shortly after the assembly, contigs with sizes smaller than 350 pb were removed to reduce the number of artifacts in the assembly. Next, Prokka (Seemann, 2014) was used to identify the coding regions used later to identify antibiotic resistance genes by the Comprehensive Antibiotic Resistance Database (CARD) and Resistance Gene Identifier (RGI) databases (Alcock et al., 2020), in addition to the identification of resistance genes to biocides and heavy metals using the BacMet software (Pal et al., 2014). After that, the contigs were submitted to the public database of the metagenomic data analysis platform MG-RAST (Meyer et al., 2008), identifying functional genes using the SEED Subsystems database, with an alignment of 90%, a size of 20 bp, and an *e*-value of 10^−5^.

### Statistical analysis

The normality analysis of the variables was performed using the Shapiro–Wilk normality test, with a significance of α = 0.05. The difference between the means was evaluated with the ANOVA and Kruskal–Wallis tests, while the Bonferroni correction was performed using the Tukey and Mann–Witney tests. Principal component analysis (PCA), ANOSIM, and cluster analysis were performed using the PAST program (Hammer et al., 2001). The Infogrames were developed with the Orange Data Mine tool (Demšar et al., 2013). The k-means method was used to create a heat map that picked 50 program clusters.

## Results

### Physicochemical analysis

The soils collected showed significant differences in terms of pH, with values varying between 5.7 and 6.9; the 2-years forest and culture sites were considered to have “high acidity” and “very low acidity,” which was different among all sites (Tukey's test, *p* < 0.05), except for pasture and 14-years sites (Tukey; *Q* = 0.471; *p* = 0.986). The levels of cations in the different sites were similar for potassium and calcium. However, for magnesium, significant differences were observed between sites about everything on the 2-years site and the other sites (Tukey's test, *p* < 0,05). The concentrations of micronutrients and heavy metals were found in the following decreasing order Fe≫ Cr > Mn > Ni > Zn > Co > Cu > As, with the highest retention of these elements in the 14-years-old culture sites. However, the iron content was higher in the forest site, without significant differences between the other sites; the arsenic, cobalt, chromium, copper, and nickel contents were also similar; in the case of manganese, there were differences between the sites with the highest content in the pasture site e 2 years (Tukey's test; *Q* = 5,516; *p* = 0.019), zinc showed significant differences with higher values in the 14-years-old culture and pasture sites, the 14-years-old site with forest and 2 years (Tukey's test, *p* < 0.05), pasture with 2 years (Tukey's test; *Q* = 5,821; *p* = 0.014; Table 1). Lead was identified only at the 14-years-old site (0.04 ± 0.07 mg kg^−1^), and mercury was found at 2-years pasture and culture sites up to 0.02 mg kg^−1^ but was not identified. Of cadmium anywhere.

**Table 1:** Chemical characteristics were obtained from soil analysis, pH values, micronutrient contents, and heavy metals.

	**pH**	**K^+^**	**Ca^2+^**	**Mg^2+^**	**As**	**Co**	**Cr**	**Cu**	**Fe**	**Mn**	**Ni**	**Zn**
		**cmol**_**c**_ **dm**^**−3**^			**mg Kg** ^**−1**^							
Forest	5.73 ± 0.06	0.1 ± 0.02	4.59 ± 0.91	0.94 ± 0.09	0.34 ± 0.44	0.75 ± 0.34	70.71 ± 5.27	0.27 ± 0.06	86.67 ± 15.04	17.83 ± 4.76	2.45 ± 2.21	0.9 ± 0.1
Pasture	6.2 ± 0.2	0.34 ± 0.27	4.21 ± 0.4	1.00 ± 0.08	0.51 ± 0.16	0.62 ± 0.23	72.46 ± 11.33	0.47 ± 0.15	85.67 ± 44.64	25.47 ± 8.55	2.55 ± 0.95	1.47 ± 0.4
2 years	6.93 ± 0.11	0.12 ± 0.01	4.47 ± 0.21	1.68 ± 0.25	0.37 ± 0.05	0.43 ± 0.15	70.81 ± 8.18	0.33 ± 0.06	61.33 ± 9.61	9.5 ± 0.46	1.88 ± 1.38	0.67 ± 0.15
14 years	6.17 ± 0.06	0.4 ± 0.11	3.71 ± 0.18	1.k01 ± 0.1	0.69 ± 0.38	0.71 ± 0.48	88.39 ± 1.83	1 ± 0.56	67 ± 13.74	15.43 ± 2.14	4.62 ± 3.72	1.9 ± 0.17
*p*-value	1.6 ×10^−5^*	0.085	0.241	0.001*	0.359	0.648	0.055	0.055	0.527	0.027*	0.529	0.001*

K^+^, potassium cation; Ca^2+^, calcium cation; Mg^2+^, magnesium cation; As, arsenic; Cd, cadmium; Co, cobalt; Cr, chromium; Cu, copper; Fe, iron; Hg, mercury; Mn, manganese; Ni, nickel; Pb, lead; Zn, zinc.

^*^p-value < 0.05.

### Microbial diversity

**Figure 1:**
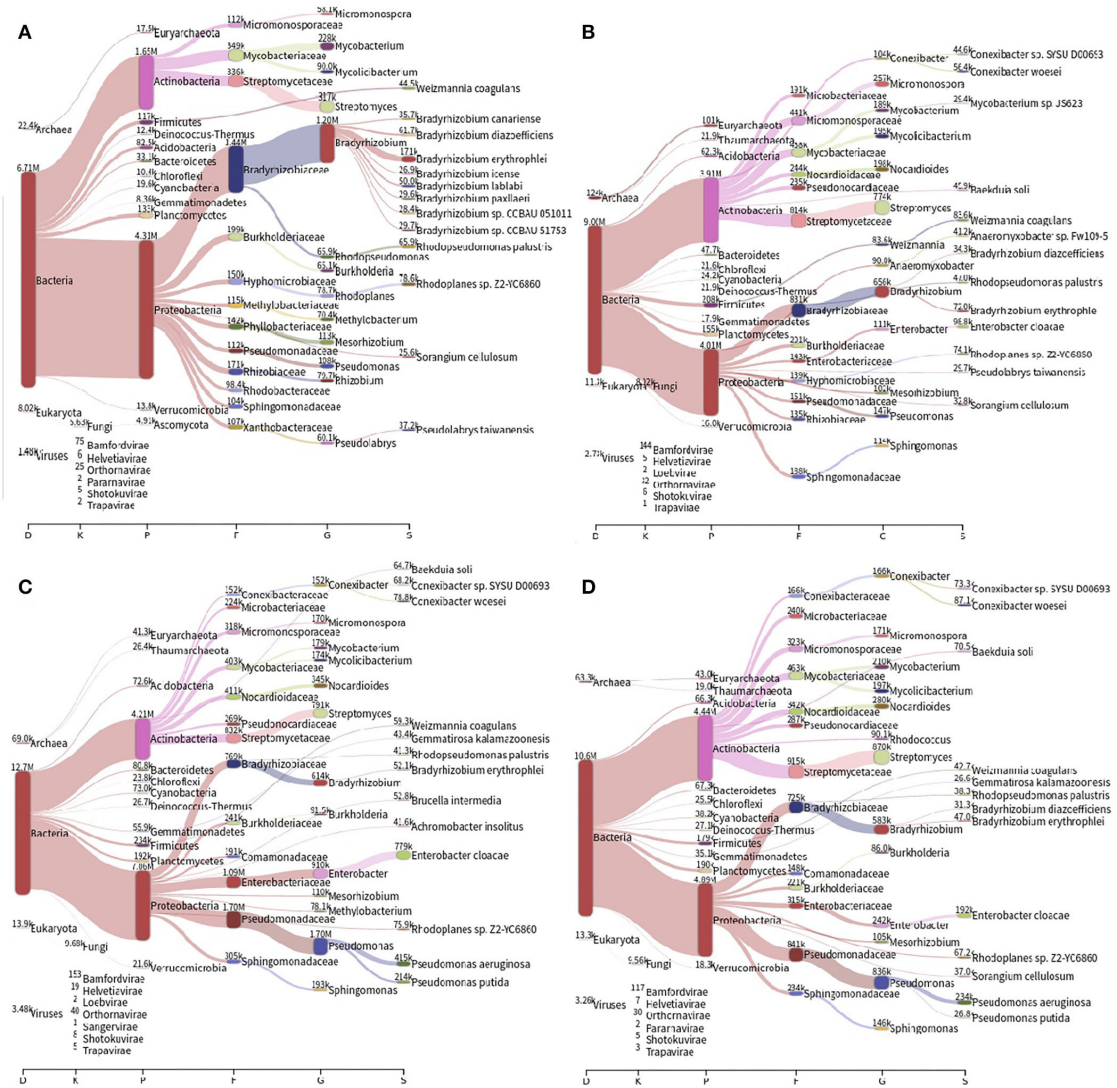
Distribution of the different bacterial taxonomic groups identified in the soils of **(A)** forest, **(B)** pasture, **(C)** 2-years soybean monoculture, and **(D)** 14-years soybean monoculture. The number of readings is classified as K, kingdom; P, phylum; F, family; G, genus; S, species.

The most abundant bacterial phyla present in different soils are Proteobacteria and Actinobacteria. The prevalence of bacterial species was *Bradyrhizobium* sp. and *Rhodoplanes* sp. in the forest samples, and *Conexibacter* sp., *Enterobacter cloacae, Pseudomonas aeroginosa*, and *P. putida* in the other soils, in addition to *Bradyrhizobium* sp., and *Rhodoplanes* sp. (Figure 1). Shannon's alpha diversity index is high in all soils, with similar values between them (ANOVA; *F* = 0.44; *p*-value = 0.73).

### Abundance of functional genes

**Figure 2:**
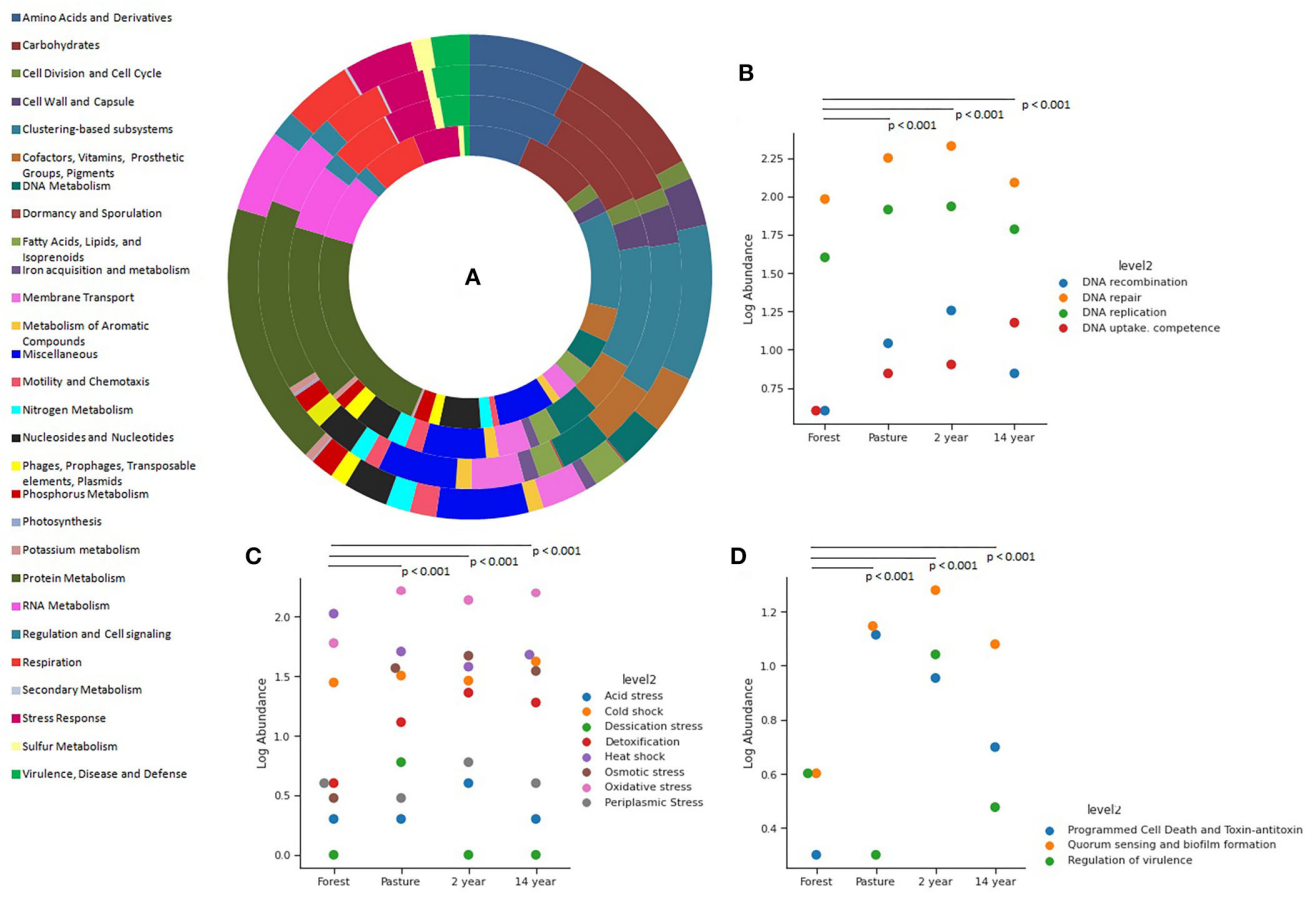
Distribution of the functional classes of genes from different soils, identified from the SEED Subsystem database: **(A)** First level of genes: from the inside out is the forest, pasture, 2-years, and 14-years soybean sites. The classification at the second level of the genes of **(B)** DNA metabolism, **(C)** stress response, and **(D)** cell regulation and signaling.

The relative abundance of functional genes according to the grouping of the SEED Subsystems database at its first level demonstrates similarities between the samples (Kruskal–Wallis; χ^2^ = 5.823; *p*-value = 0.12), with the highest relative number of genes being from functional classes related to protein, carbohydrate, and amino acid metabolism, RNA synthesis, stress response, transport of elements, dormancy, among others (Figure 2A).

The number of gene groups associated with adaptation and stress processes at a second level was higher in crop and pasture soils. Furthermore, it is possible to observe that the abundance of genes associated with DNA metabolism showed significant differences between soils (Kruskal–Wallis; χ^2^ = 36.61; *p*-value = 1.27 × 10^−5^), with a higher overall proportion of genes involved in repair and replication mechanisms (Figure 2B).

The composition of genes associated with the stress response process showed significant differences between soils (Kruskal–Wallis; χ^2^ = 46.33; *p*-value = 1.29 × 10^−11^): In forest soil, the genes responsible for the stress response were more prevalent than the heat shock response genes, constituting a response to oxidative stress and cold shock, respectively; in monoculture soils, in addition to the previously mentioned genes, there were genes for osmotic stress response and detoxification; and in pasture soil, a dominance of genes responding to oxidative stress and desiccation was observed, followed by genes from the functional classes mentioned above (Figure 2C).

Similarly, the number of genes associated with cell regulation and signaling showed significant differences between the samples (Kruskal–Wallis; χ^2^ = 38.37; *p*-value = 2.36 × 10^−8^), with crop and pasture soils demonstrating a prevalence of genes for quorum sensing, biofilm formation, programmed death, and toxin-antitoxin, while in forest soil there is a prevalence of genes for quorum sensing, biofilm formation, and virulence regulation (Figure 2D).

### Mobile elements and pathogenicity islands

**Figure 3:**
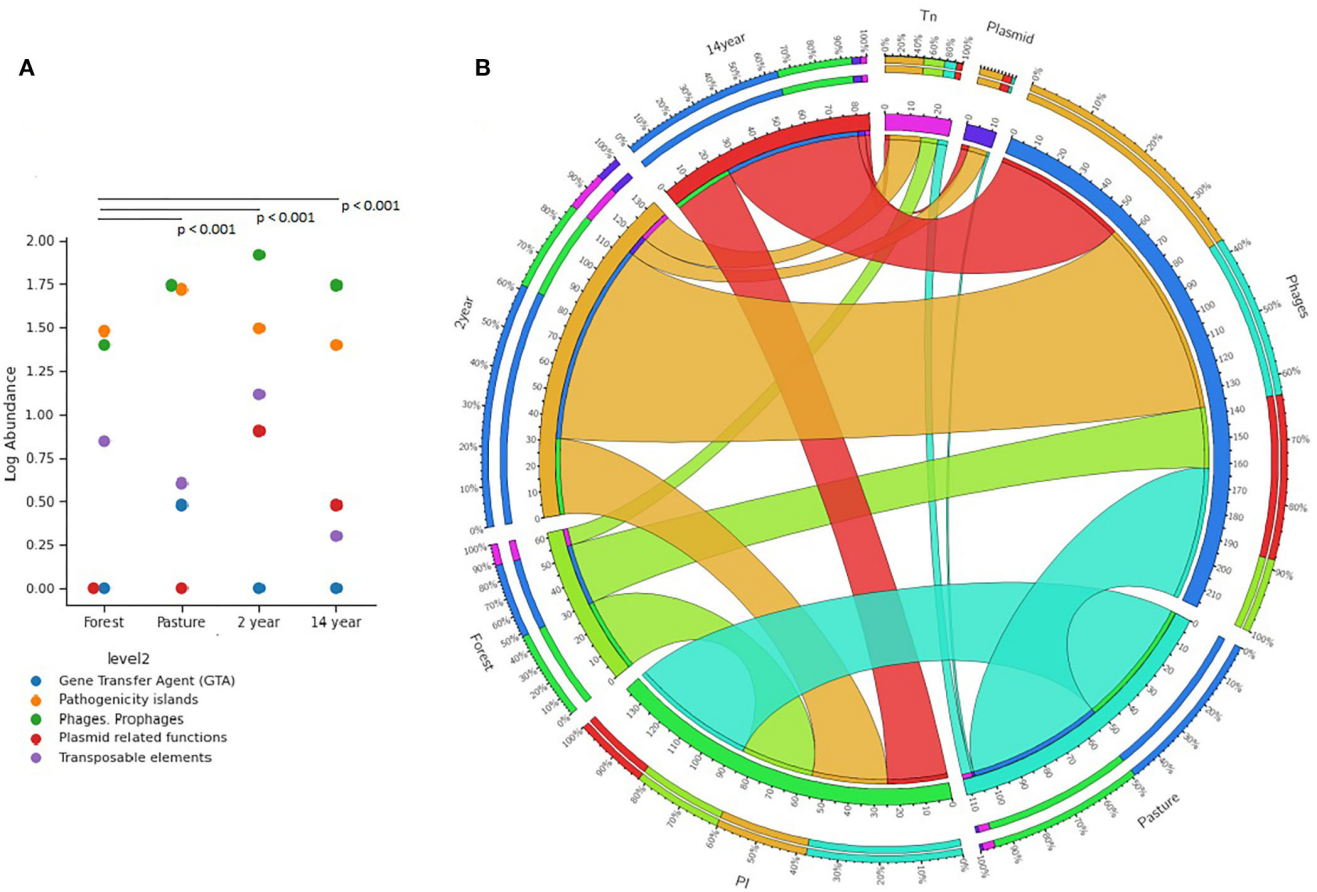
Distribution of the relative abundance of genes identified from the SUB Systems database of the MG-RAST platform for gene transfer agents (GTA), pathogenicity islands (PI), phages and prophages, plasmids, and transposons (Tn); **(A)** distribution in the different soils, the *p*-values obtained from Mann–Witney were corrected by Bonferroni analysis; **(B)** graphic representation of this distribution in circles.

The number of genes related to pathogenicity islands and mobile elements (phages, prophages, plasmids, transposons, and transfer genes) showed significant differences between soils (Kruskal–Wallis; χ^2^ = 13.39; *p*-value = 0.001). The prevalence of genes associated with phages and prophages increased in tilled soils, while the amounts in forest and pasture soils decreased. Similar to the previous one, the genes related to the most prevalent pathogenicity islands in different soils, especially in pasture samples (Figures 3A,B).

### Antibiotic and biocide resistance genes

**Figure 4:**
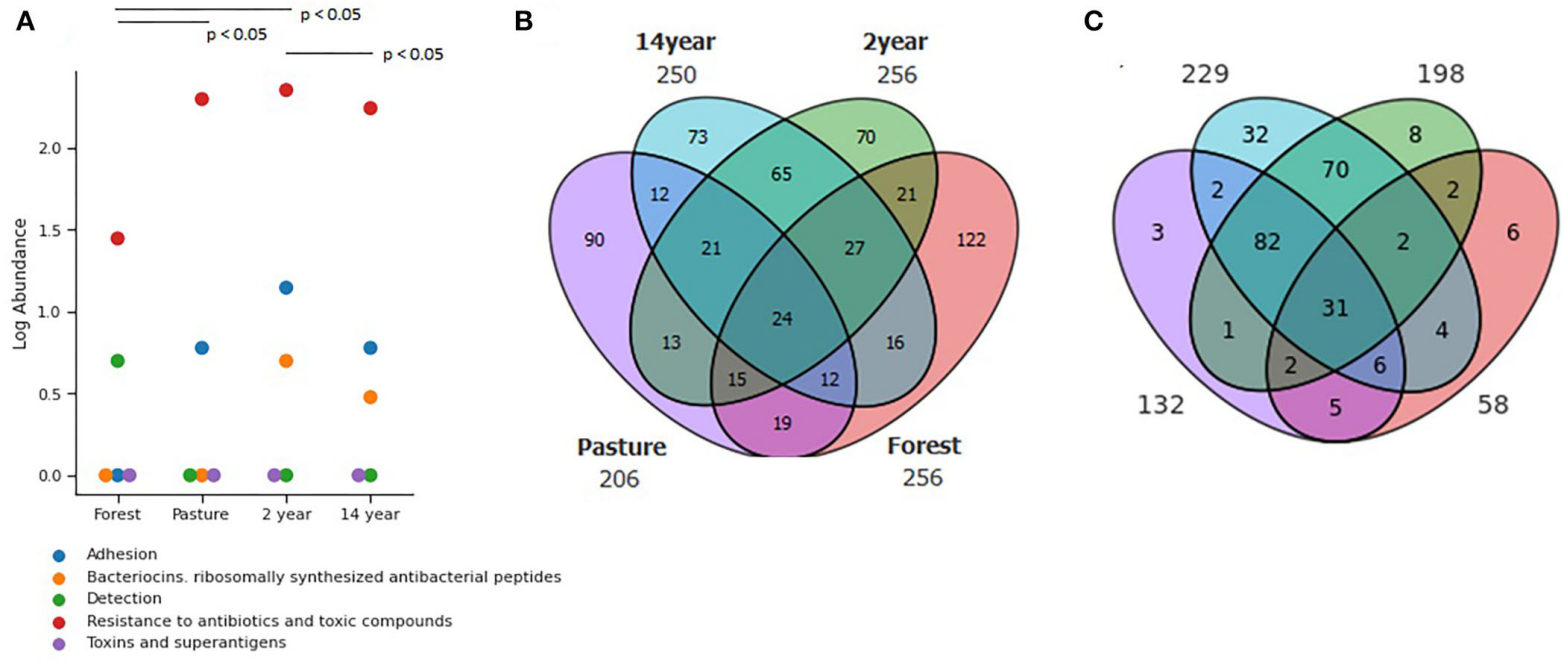
Distribution of **(A)** Functional genes in different soils identified from the SEED Subsystems database, demonstrating clustered genes of virulence, disease, and defense at its second level. Venn diagram of identified genes from **(B)** Antimicrobial resistance identified from CARD database and **(C)** Biocide resistance in different soils identified from BacMet database.

A large number of resistance genes were identified in different soils, with significant differences (Figure 4A) between pasture and crop soils (Kruskal–Wallis; χ^2^ = 72.22; *p*-value = 7.98 × 10^−18^). In this context, up to 600 distinct genes were identified in the analyzed samples, among which 43% displayed multidrug resistance.

The presence of antibiotic resistance genes was observed at all sites. Comparatively, 24 genes are shared between the sites. As for the total number of genes identified in each soil, the forest had 122 genes, followed by pasture with 90 genes and crop soils with up to 73 genes (Figure 4B).

Regarding biocide resistance genes, a total of 256 genes were identified, of which 53% showed multi-resistance, especially to different heavy metals. A higher prevalence was detected in soils with 2–14 years of monoculture and pasture, making it possible to observe 31 genes in common in all analyzed soils. The 14-years-old soil crop had the highest number of unique biocide resistance genes, with 32 genes (Figure 4C).

**Figure 5:**
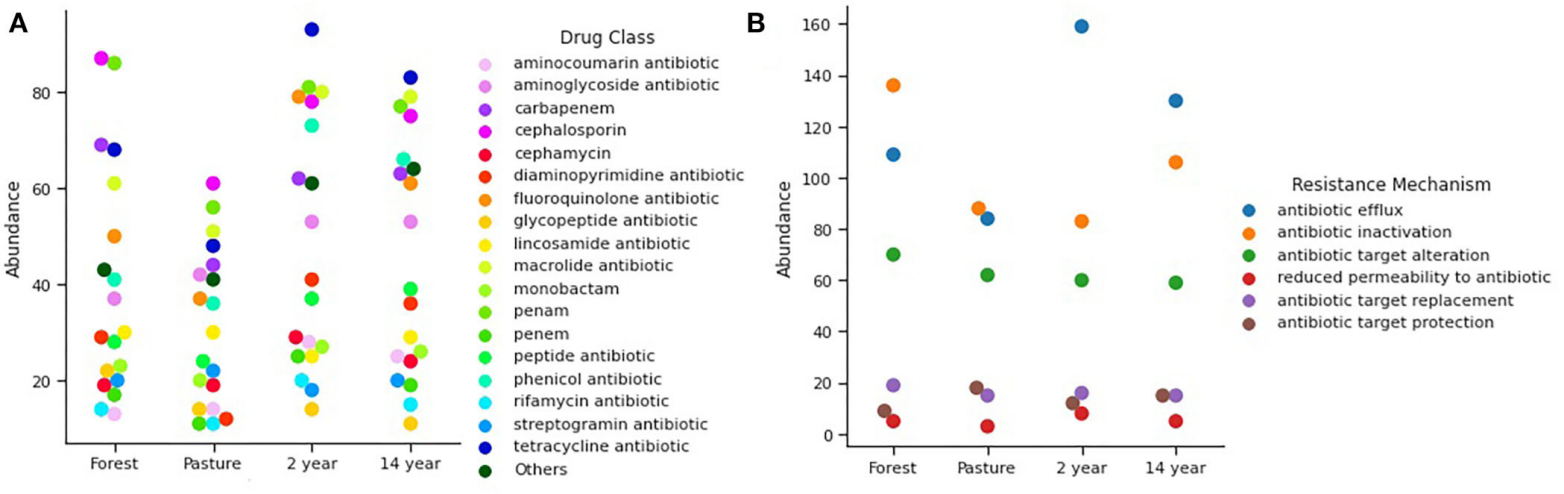
Relative abundance of **(A)** antibiotic classes according to the genes found for antimicrobial resistance. **(B)** Mechanisms of resistance found according to the genes identified.

The relative abundance of antibiotic resistance genes in the analyzed samples was of 32 different antibiotics, without showing significant differences between the soils (Kruskal–Wallis; χ^2^ = 1.242; *p*-value = 0.742), among which the resistance to classes of antibiotics. The most prevalent was resistance to tetracycline, cephalosporin, penam, fluoroquinolone, chloramphenicol, carbapenem, macrolide, and aminoglycoside (Figure 5A). Regarding the occurrence of the resistance mechanisms identified, there is a similarity between the soils (ANOVA; *F* = 0.076; *p*-value = 0.972), with the most common mechanisms being flow pumps, inactivation of antibiotics, and alteration of targets. Among these, efflux pumps were more abundant in 2- and 14-years-old monoculture soils, while in forest soil, the most abundant was antibiotic inactivation (Figure 5B).

**Figure 6:**
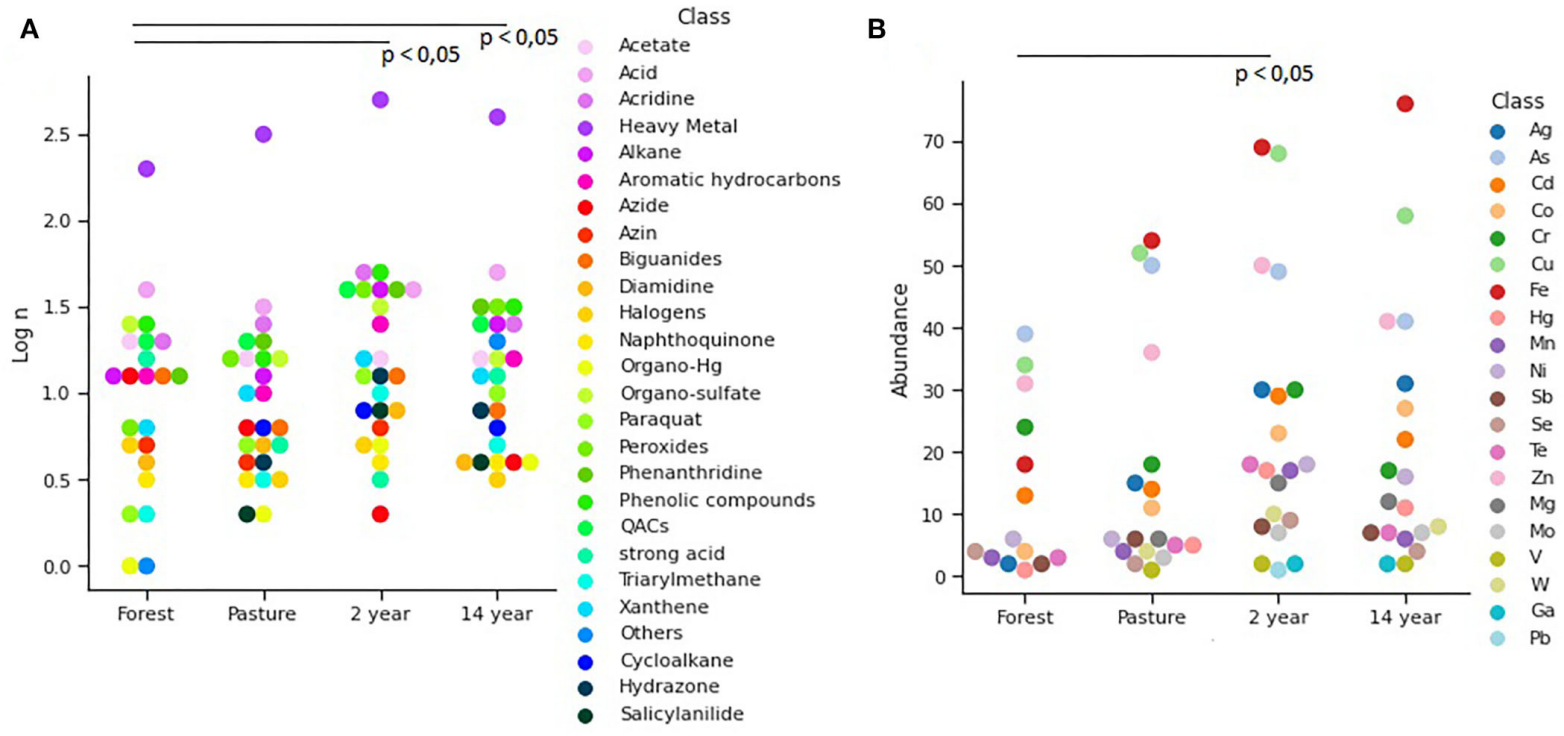
The distribution of **(A)** biocides and **(B)** heavy metals identified from the BacMet database showed significant differences (*p* < 0.05) between soils.

As for biocide resistance, 94 genes associated with resistance to different chemical compounds were identified, and it was possible to observe significant differences between the genes found in each soil (Kruskal–Wallis; χ^2^ = 9.405; *p*-value = 0.023). Among them, heavy metals were the most abundant, followed by phenolic compounds, quaternary ammonium (QACs), and aromatic hydrocarbons, which are more prevalent in soils (Figure 6A). There was also evidence of resistance to heavy metals and a significant difference between the samples (Kruskal–Wallis; χ^2^ = 11.11; *p*-value = 0.011), with pasture and crop soils showing the greatest abundance of iron resistance genes, iron, copper, arsenic, and zinc (Figure 6B).

The distribution of resistance mechanisms was similar between soils (Kruskal–Wallis; χ^2^ = 7.648; *p*-value = 0.053), with the efflux pump and enzymatic inactivation being the most frequent.

**Figure 7:**
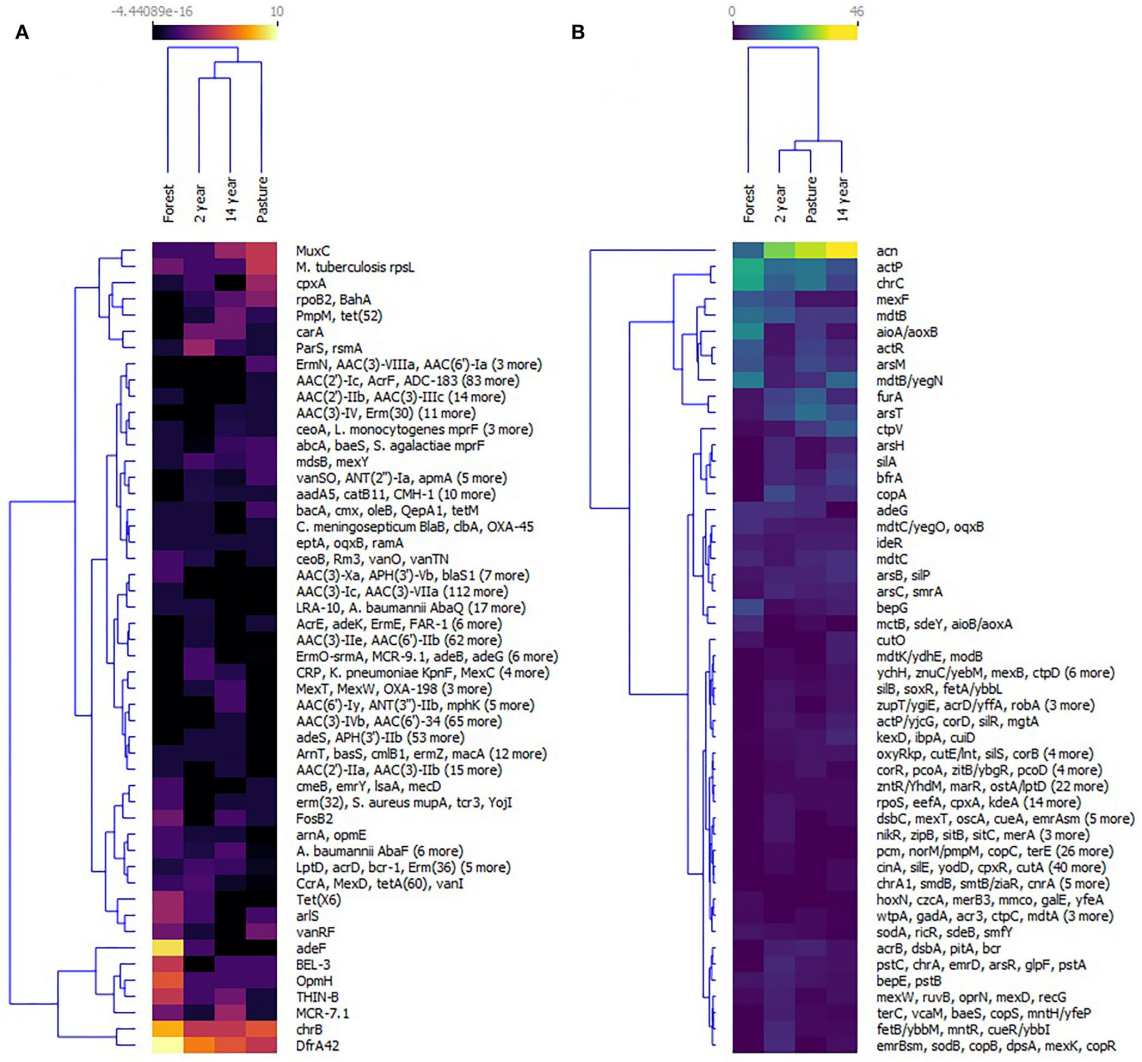
Heat map of **(A)** antibiotic resistance genes and **(B)** biocides resistance genes identified at collection sites—grouped using the k-means algorithm.

Similarities between crop and pasture soils were observed during the distribution of the groups of antibiotic and biocide resistance genes according to their relative abundance. The diversity of these genes was observed, especially in the forest soil. The most prevalent genes in this soil were *dfrA42* (trimethoprim-resistant dihydrofolate reductase), *chrB* (23S ribosomal RNA methyltransferase), *MCR7.1* (Phosphoethanolamine transferase), *THIN-B* (Beta-lactamase), *OpmH* (resistance-nodulation-cell division (RND, efflux pump), *BEL3* (beta-lactamase) and *adeF* (RND). In crop soils, the most abundant genes were *MuxC* (RND), *rpsL* (mutations that confer resistance to streptomycin), *cpxA* (RND), *rpoB2* [rifamycin-resistant RNA polymerase beta subunit (rpoB)], *tet(52)* (Tetracycline inactivating enzyme), *carA* (ABC-F ATP-binding cassette ribosomal protection protein), *ParS* [RND; outer membrane porin (Opr)] and *rsmA* (RND) (Figure 7A).

Likewise, the analysis of the relative abundance of biocide resistance genes showed a similar grouping between cropland and pasture soils, with the composition of the genes with the highest prevalence being *acn* (Aconitato hydratase) in all soils, followed by genes *actP* (Copper transporter P-type ATPase), *chrC* (Fe-dependent superoxide dismutase), *mexF* (RND; MexF permeases protein), *mdtB* (MdtB multidrug-resistant protein), *aioA/aoxB* (Arsenite oxidase subunit), *actR* (Regulator protein ActR acid tolerance), *arsM* (Arsenite S-adenosylmethyltransferase), *mdtB/yegN* (MdtB multidrug-resistant protein), *FurA* (Transcriptional regulator), and *arsT* (Thioredoxin reductase) (Figure 7B).

### Association of genes and chemical characteristics

**Figure 8:**
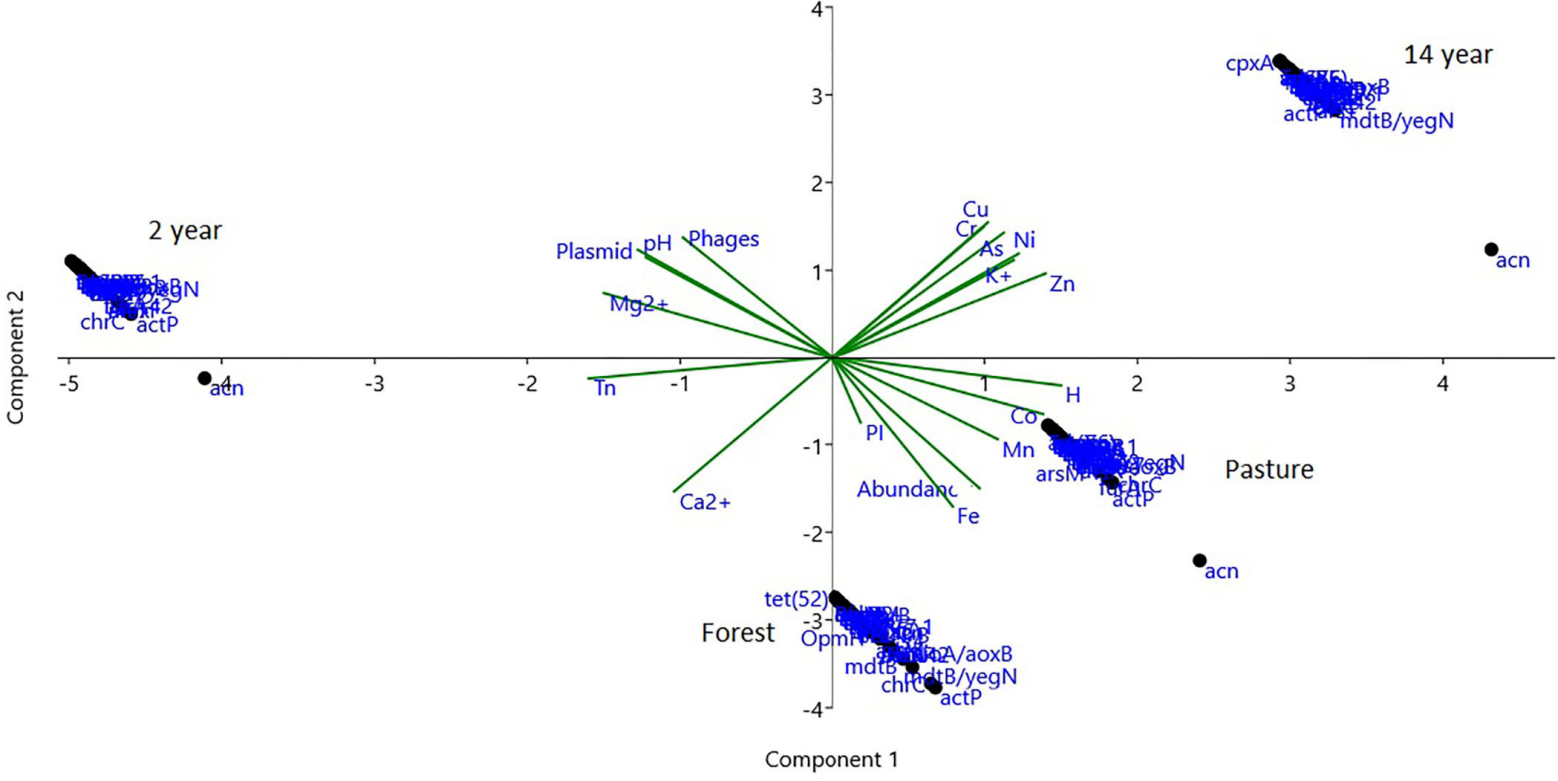
PCA analysis of genes with a greater abundance of antibiotic and biocide resistance genes, Shannon's alpha diversity index (H) also of plasmid mobile elements, phages, and transposons (Tn), pathogenicity islands (PI) with the chemical characteristics of pH, cations, and heavy metals.

Principal component analysis (PCA) showed an accumulated variability of 99% in three components, represented by the first component (PC1) with 53% and the second component (PC2) with 34%, explaining the variability of the data. Further, the clustering of resistance genes with higher relative abundance was different in all soils (ANOSIM; *R* = 0.984; *p*-value = 0.000), especially in the 2-years monoculture soil. Thus, an association of the variables of heavy metal content was observed in the soils of 14-years-old culture and pasture, while the mobile elements identified are associated with magnesium cation, and the pH is relevant to the 2-years-old culture soil (Figure 8).

**Figure 9:**
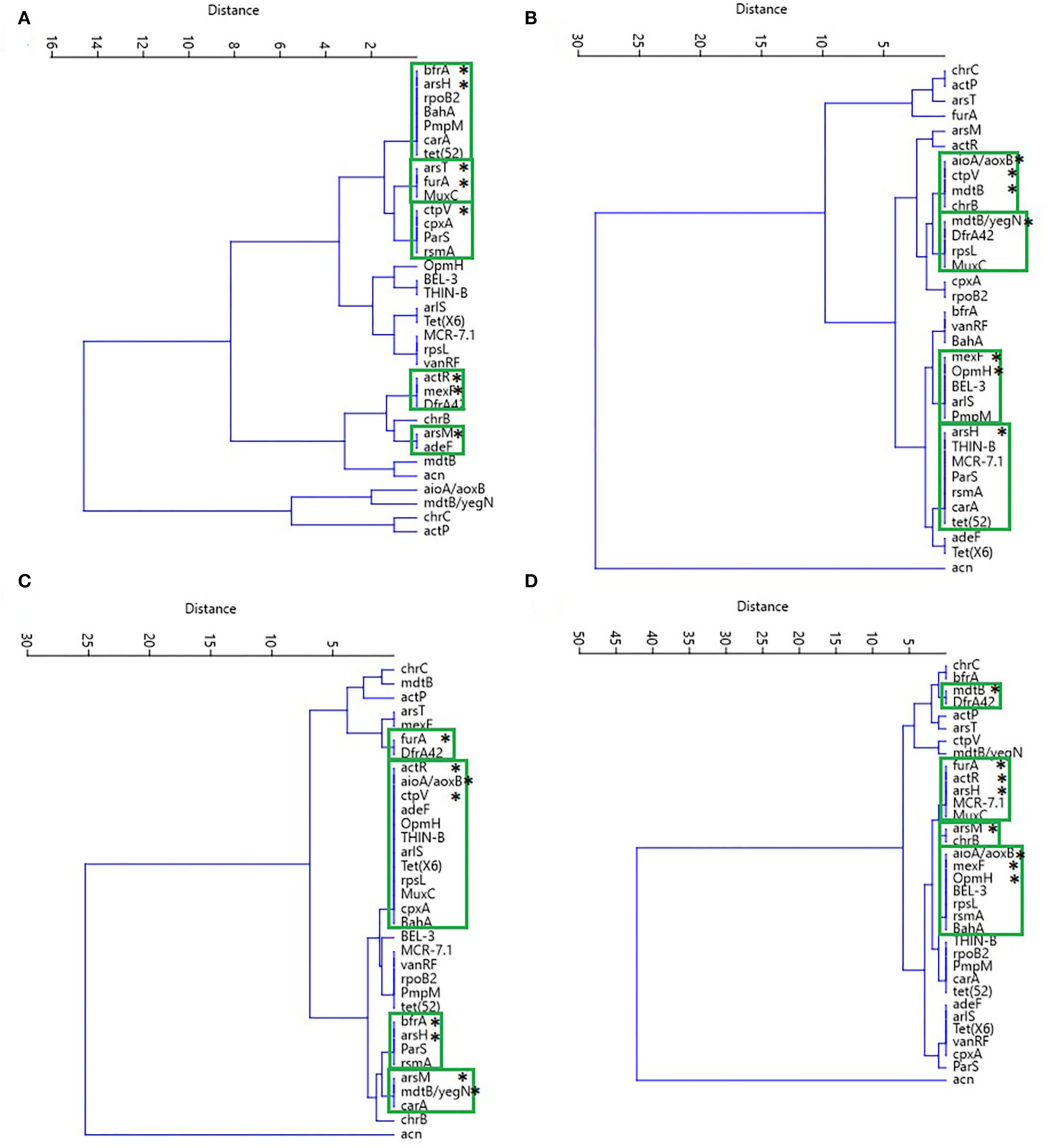
Cluster analysis performed by the UPGMA algorithm and the Euclidean similarity index of the most abundant resistance genes to antibiotics and biocides and the characteristics analyzed in soils from **(A)** forest, **(B)** pasture, **(C)** 2-years crop, and **(D)** culture of 14 years. In the green boxes are grouped the genes of resistance to antibiotics and biocides (*).

The resistance genes for antibiotics and biocides with the highest relative abundance according to the variables previously used were grouped into different dendrograms for each soil, and the most prevalent genes were in the formation of the outgroups. In forest soil, biocide resistance genes (*aioA/aoxB, mdtB/yegN, chrC*, and *actP*) are related to heavy metals, strong acids, and organic compounds, while pasture and crop soils presented the *acn* gene as an outgroup and iron resistance (Figure 9).

In the clades that presented the combinations of biocide and antibiotic resistance genes, there was a higher prevalence of resistance genes for iron and arsenic in the forest soil, in addition to the resistance to the fluoroquinolone, tetracycline, aminoglycoside, macrolide, and chloramphenicol classes of antibiotics (Figure 9A). In the pasture soil, the genes for triclosan and resistance to the tetracycline, aminoglycoside, fluoroquinolone, and monobactam classes of antibiotics were predominant (Figure 9B). In the 2-years-old monoculture soil, the prevalence occurred in the iron and arsenic resistance genes, as well as for the tetracycline, aminoglycoside, macrolide, and fluoroquinolone antibiotic classes (Figure 9C). In contrast, in the 14-years-old monoculture soil, the following were identified as resistance genes to arsenic, iron, chromium, triclosan, and the macrolide and monobactam classes of antibiotics (Figure 9D). Furthermore, multi-resistance genes to antibiotics, such as *ParS* and *carA*, are highlighted in different sites.

## Discussion

### Chemical characteristics and bacterial communities

The expansion of the agricultural frontier by using fire occurs by eliminating the vegetation cover of the soils, which favors changes in chemical components, as well as the increase in the levels of heavy metals, such as nickel, manganese, cobalt, cádmium, and arsenic (Stefanowicz et al., 2012; Popovych and Gapalo, 2021). However, the chemical characteristics of Amazonian soils vary according to the type of terrain and region, with specific contents of native chemical compounds (Costa et al., 2017).

In this work, it was possible to observe the variation of chemical characteristics in all analyzed soils, with higher pH, cations, and some heavy metals analyzed in monoculture soils. On the forest site, higher iron content was found, which reflects the presence of this element natively in this place. The accumulation of heavy metals can decrease the quality of soils, which can affect the health of consumers in the long term through bioaccumulation in vegetables (Salam et al., 2020).

Furthermore, although changes in the ecosystem can significantly influence the composition of soil bacterial communities (Meng et al., 2019; Salam et al., 2020), in this work, the diversity index values did not show significant changes due to chemical variations in soils, as reported by Thomas et al. (2020), but the change in the structure of microbial communities was observed, causing enrichment and recruitment of beneficial microorganisms for cultivation (Zhou et al., 2021).

In general, the bacterial communities in Amazonian soils are mainly composed of the phylum Proteobacteria and Acidobacteria, Actinobacteria, Bacteroidetes, and Firmicutes (Chodak et al., 2013; Shen et al., 2019; Thomas et al., 2020). However, the results of this work reveal a different composition between the sampled environments, with the forest floor showing the prevalence of different species of *Bradirhyzobium* sp.; it was in contrast to crop and pasture soils, in which *Enterobacter cloacae* and different species were relevant species of *Pseudomonas* sp., these data can be explained by the very conservation of soils for crops, which favor the development of these types of bacteria (Forsberg et al., 2012; Armalyte et al., 2019; Macedo et al., 2021; Yasir et al., 2022).

### Analysis of the composition of functional genes and soil resistome

The analysis of the diversity of functional genes identified in all soils reveals similarities in composition, but the relative abundance of these was different for the samples from each site, with the highest values found in genes associated with the metabolism of proteins, carbohydrates, amino acids, synthesis of RNA, stress response, and transport of elements, and it is possible to observe a more significant presence of these genes in soils where vegetation cover was removed, as reported in other works (Castañeda and Barbosa, 2017; Yasir et al., 2022).

The different communities of microorganisms that coexist in a given ecosystem are subject to the pressure of different stress factors inherent to their environment, such as climate and environmental change, and the presence of anthropic contaminants, among others. Soil microbial communities are generally sensitive to these disturbances from abiotic factors (drought, low/high temperature, salinity, and acidic conditions, light intensity, submersion, anaerobiosis, and nutrient deficiency) and biotic factors, but these microorganisms have several mechanisms that allow their adaptation in the environment, being able to maintain their main physiological functions (Pérez-Valera et al., 2020).

Thus, in this work, genes associated with the response to these environmental disturbances were identified, as well as genes involved with DNA repair mechanisms, which are more significant than replication genes, especially in soils of culture and pasture. In the case of genes associated with the response to defense mechanisms against abiotic stress, the composition of the forest soil was different from that of other soils, which possibly reflects the consequences of the removal of vegetation cover, in addition to the possibility that the type of forest management soil alters genetic diversity (Meena et al., 2017).

Soils without forests showed a prevalence of genes involved in quorum sensing and biofilm formation, which may be advantageous for the local vegetation due to the nutrient absorption capacity (Bhagat et al., 2021) or probably due to the application of agro-compound chemicals. Microorganisms show their ability to biodegrade in response to stress (Chen et al., 2021). Different bacterial communities have different mechanisms that help them recover quickly, returning to their microbial functions and activities as soon as the stress factor ends (Schimel, 2018; Bremer and Krämer, 2019; Schnecker et al., 2021).

Likewise, the conditions of these environments can promote the selection of genetic components and their dissemination through mobile genetic elements (MGE), which can carry pathogenicity genes and antibiotic and biocide resistance genes (Martins and Rabinowitz, 2020). Thus, in crop soils, the presence of pathogenicity islands, phages, prophages, transposons, and plasmids was predominant, especially in the soil after 2 years of cultivation. As for antibiotic and biocide resistance genes, there was a more significant amount in pasture and crop soils, although the forest soil showed a greater diversity of antibiotic resistance genes. In crop soils, the resistance genes were more diverse to biocides.

Natural forest soils are a significant source of antibiotic ARG, and their large-scale distribution is regulated by the diversity of microorganisms and herbaceous plants (Hu et al., 2018; Qian et al., 2021). In this way, native ARGs in different soils can be shared with plants, becoming a potential route for the transfer of these genes to the human microbiome and the potential for transferring pathogens through the food chain (Zhang et al., 2019).

However, it remains unclear whether traditional agricultural activities affect the soil resistome (Qian et al., 2021), although data on sewage sludge contamination constitutes a vast repository of antibiotic, biocide, and MGE resistance genes that can affect the soil resistome (Markowicz et al., 2021).

The native antibiotic resistance profile of soil is varied but always presents high gene diversity (Qian et al., 2021; Zheng et al., 2021), as shown in forest soil. As for the profile of the resistome of the crop soils, a strong association was not found; it is possibly affected by the addition of compounds, such as fertilizers, and manure, among others (Martins and Rabinowitz, 2020; Macedo et al., 2021).

Analysis of soil resistance to antimicrobials and biocides shows that the most frequently used resistance mechanisms are efflux pumps and antibiotic inactivation, which reflects the evolution of microorganisms that have adapted to survive in ecosystems regardless of certain conditions. Changes likely serve as buffers for ecological niches (Walsh and Duffy, 2013; Armalyte et al., 2019). In this work, levels of different heavy metals were identified in the soil after 14 years of culture, while in the soil, after 2 years of culture, a more significant variation in pH, magnesium cation content, and the different EGM components were highlighted. Bivalent cations such as magnesium influence soil quality and plant development, in addition to being a metallic cofactor for different enzymatic processes for microorganisms (Sissi and Palumbo, 2009; Gerendás and Führs, 2013; Shin et al., 2014).

However, the results of this work suggest that, even in areas with low levels of heavy metals (Arsenic, copper, zinc, cobalt, and chromium), there is an induction to the selection of resistance to these elements, which could also be selecting resistance genes. Antibiotic resistance genes are ubiquitous. In addition, human activities also influence the selection of the composition of microbial communities, resistance genes, mobile elements, and other markers (Thomas et al., 2020).

Additionally, the presence of mobile genetic elements increases the spread of resistance genes through HGT, which can also be affected by environmental conditions and other stressors (Shen et al., 2019). In this study, it was possible to observe the presence of resistance genes to antimicrobials and widely used biocides, which makes the presence of MGEs in soils an indication of the potential of these molecules to promote the conjugative transfer of resistance at concentrations below the minimum inhibitory concentration, as previously stated. Identified in another work on tetracycline resistance, which is a potent inducer of conjugation (Jutkina et al., 2018). Likewise, nickel exposure has been reported to increase the horizontal transfer potential of ARG (Hu et al., 2017); in our work, the high levels of iron found in the forest could be a factor in the selective pressure on native microbial communities; this pressure would increase with the change of ecosystems for the development of pasture and culture and would be reflected in the diversity and abundance of resistance genes, and the presence of mobile elements.

### Co-selection of resistance genes

The metagenomic analysis allows a correlation between antibiotic and biocide resistance genes, providing additional information on heavy metal-induced ARG co-selection (Wang et al., 2020). Cross-resistance and co-resistance are the main drivers for ARG co-selection. However, other mechanisms are also involved in this phenomenon, such as the presence of a single regulatory gene for several resistance genes (Maurya et al., 2020).

In the results of this work, a correlation between resistance to antibiotics and biocides was identified between the genes of resistance to the classes of antibiotics: tetracycline, aminoglycoside, macrolide, and fluoroquinolone and the metals iron, arsenic, and copper.

Amazon deforestation has increased the amount of ARG in the soil and, like other anthropogenic disturbances, can exert selective pressure on microbial communities, expanding soil resistome and increasing microbial diversity in response to deforestation, along with changes in soil chemical properties, such as pH and the presence of aluminum (Lemos et al., 2021). This work observed no significant change in ARG diversity or abundance of these genes after long-term continuous cultivation. An increase in the diversity and abundance of biocide resistance genes was evidenced.

The analysis of resistance genes among the soil samples of this study suggests the development of resistance to antibiotics and biocides under different selection pressures in different environments. However, our data are limited by the number of sampling at the sites and the lack of longitudinal analysis. Despite these limitations, our results can be applied to other related studies, such as a comparative analysis of the soil microbiome and other environments to explore the diversity of ARG between different sites. For studies about the transfer of these genes from the environment to the human commensal microbiota, more sampling sites should be considered, in addition to the collection at different times to identify the soil microbiome and its resistome, thus making it possible to obtain more information about the development and ARG broadcasts. Even so, an analysis beyond genes, such as identifying ARG transcripts by metatranscriptomic sequencing, can provide a more comprehensive understanding of their dynamics (Zheng et al., 2021).

## Conclusion

The analysis carried out on the different soils highlights the differences in chemical composition, bacterial communities, and genetic composition between different cultivation soils. These ecosystems presented a differentiated abundance of genetic composition and mobile genetic elements in other sites. Above all, the sites eliminated the vegetal cover.

On the other hand, it was demonstrated that antibiotic resistance genes were diverse in the forest site and more abundant in the crop soils. In contrast to the genes of resistance to biocides, especially for heavy metals, they were diverse and more abundant in the cultivation sites. As for antibiotic resistance, the most frequent were the antimicrobials aminoglycosides, macrolides, tetracyclines, and fluoroquinolones. In contrast, the most prevalent resistance to biocides is related to iron in different sites with high levels. Eliminating vegetative cover may alter the selection of other animals and genes that have the potential to affect human health.

## Data availability statement

The datasets presented in this study can be found in online repositories. The names of the repository/repositories and accession number(s) can be found at: NCBI-PRJNA841936.

## Author contributions

OC, MP, ACB, RR, and ARC contributed to conception and design of the study. ES, AO, ACR, and OC performed the statistical analysis manuscript. All authors contributed to manuscript revision, read, and approved the submitted version.

## Funding

Funded National Research Council (CNPq), Alliance Program for Education and Training—PAEC-OEA-GCUB 2017, within the scope of the Cooperation Agreement between the Organization of American States (OAS) and the Coimbra Group of Brazilian Universities (CGUB) and L'Oréal Brasil-UNESCO-ABC For Women in Science.

## Conflict of interest

The authors declare that the research was conducted in the absence of any commercial or financial relationships that could be construed as a potential conflict of interest.

## Publisher's note

All claims expressed in this article are solely those of the authors and do not necessarily represent those of their affiliated organizations, or those of the publisher, the editors and the reviewers. Any product that may be evaluated in this article, or claim that may be made by its manufacturer, is not guaranteed or endorsed by the publisher.
